# hsa-MiR-19a-3p and hsa-MiR-19b-3p Are Associated with Spinal Cord Injury-Induced Neuropathic Pain: Findings from a Genome-Wide MicroRNA Expression Profiling Screen

**DOI:** 10.1089/neur.2021.0011

**Published:** 2021-09-14

**Authors:** Liang Ye, Leslie R. Morse, Scott P. Falci, Julie K. Olson, Mayank Shrivastava, Nguyen Nguyen, Clas Linnman, Karen L. Troy, Ricardo A. Battaglino

**Affiliations:** ^1^ Department of Rehabilitation Medicine, University of Minnesota School of Medicine, Minneapolis, Minnesota, USA.; ^2^ Department of Neurological Surgery, Swedish Medical Center, Englewood, Colorado, USA.; ^3^ Department of Diagnostics and Biological Sciences, University of Minnesota School of Dentistry, Minneapolis, Minnesota, USA.; ^4^ Department of Physical Medicine and Rehabilitation, Harvard Medical School, Spaulding Rehabilitation Hospital, Charlestown, Massachusetts, USA.; ^5^ Department of Biomedical Engineering, Worcester Polytechnic Institute, Worcester, Massachusetts, USA.

**Keywords:** biomarker, microRNA, neuropathic pain, rehabilitation medicine, spinal cord injury

## Abstract

Neuropathic pain in spinal cord injury (SCI) is associated with inflammation in both the peripheral and central nervous system (CNS), which may contribute to the initiation and maintenance of persistent pain. An understanding of factors contributing to neuroinflammation may lead to new therapeutic targets for neuropathic pain. Moreover, novel circulating biomarkers of neuropathic pain may facilitate earlier and more effective treatment. MicroRNAs (miRNAs) are short, non-coding single-stranded RNA that have emerged as important biomarkers and molecular mediators in physiological and pathological conditions. Using a genome-wide miRNA screening approach, we studied differential miRNA expression in plasma from 68 healthy, community-dwelling adults with and without SCI enrolled in ongoing clinical studies. We detected 2367 distinct miRNAs. Of these, 383 miRNAs were differentially expressed in acute SCI or chronic SCI versus no SCI and 71 were differentially expressed in chronic neuropathic pain versus no neuropathic pain. We selected homo sapiens (hsa)-miR-19a-3p and hsa-miR-19b-3p for additional analysis based on *p*-value, fold change, and their known role as regulators of neuropathic pain and neuroinflammation. Both hsa-miR-19a-3p and hsa-miR-19b-3p levels were significantly higher in those with chronic SCI and severe neuropathic pain versus those with chronic SCI and no neuropathic pain. In confirmatory studies, both hsa-miR-19a-3p and hsa-miR-19b-3p have moderate to strong discriminative ability to distinguish between those with and without pain. After adjusting for opioid use, hsa-miR-19b-3p levels were positively associated with pain interference with mood. Because hsa-miR-19 levels have been shown to change in response to exercise, folic acid, and resveratrol, these studies suggest that miRNAs are potential targets of therapeutic interventions.

## Introduction

According to the International Association for the Study of Pain (IASP), neuropathic pain is caused by a lesion or disease of the somatosensory nervous system generally classified as central or peripheral.^1^ There are nearly 17,900 new cases of spinal cord injury (SCI) each year with over 296,000 individuals living with SCI in the United States.^2^ SCI-induced neuropathic pain represents a significant clinical challenge that affects 50–70% of men and women living with SCI. Chronic neuropathic pain is associated with significant morbidity and there are few effective treatment options. Moreover, the underlying mechanisms contributing to chronic neuropathic pain after SCI are poorly understood. Therefore, the elucidation of pathophysiological mechanisms involved in neuropathic pain associated with SCI is essential for the development of novel mechanism-based therapeutic interventions. Identification of a novel circulating biomarker of neuropathic pain would have great clinical utility as this might facilitate earlier identification and treatment of individuals likely to develop chronic neuropathic pain.

Accumulating evidence suggests that a class of small non-coding inhibitory RNAs known as microRNAs (miRNAs) play an important role in regulating pain processing in a wide range of experimental models.^3^ miRNAs are non-coding single-stranded RNA of 19–24 nucleotides with the ability to modulate a large proportion of the genome post-transcriptionally. They bind to the 3′ untranslated region (UTR), or occasionally to 5′ UTRs, of the multiple messenger RNA (mRNA) targets to which they exhibit imperfect, or sometimes, perfect complementarity. This enables one specific miRNA to inhibit expression of multiple genes.^4–6^

Specific changes in protein expression in peripheral nociceptive and central neurons are thought to contribute to the development of hyper-excitability leading to persistent chronic neuropathic pain.^1^ Because SCI-induced neuropathic pain is mediated by, among other factors, neuronal protein expression, the process can potentially be regulated by miRNAs. Many studies have been done in rodent models of neuropathy investigating changes in miRNA expression both centrally and peripherally. Most have demonstrated dysregulation in numerous miRNAs.^7–10^ It has been reported that a lesion in the periphery (sciatic nerve) can cause downregulation of specific miRNAs centrally in post-synaptic neurons of the nucleus accumbens.^3^ Further, these changes may be involved in the development of co-morbid conditions, such as anxiety and sleep disorders, that are associated with neuropathic pain.

There is a general consensus in that miRNA alterations that occur both in the central nervous system (CNS) and the peripheral nervous system (PNS) mediate or are associated with neuropathic pain. Evidence suggests that miRNAs can influence microglial activity and neuroinflammation by controlling expression levels of proteins that stimulate or inhibit microglial activation.^11,12^ Neuroinflammation is thought to contribute to neuropathic pain after SCI. To date, there is limited information on miRNAs as regulators of neuropathic pain after SCI. To begin to address this gap, we conducted untargeted genome-wide miRNA screenings to assess differential miRNA expression in plasma from healthy, community-dwelling adults with and without SCI and with or without severe neuropathic pain enrolled in ongoing clinical studies.

## Methods

### Subjects

We studied a convenience sample of participants with and without SCI who were enrolled in one of two ongoing clinical trials to improve bone health or in an observational study assessing surgical treatment for severe neuropathic pain. For all participants, data were derived from baseline testing. The first clinical trial (ClinicalTrials.gov Identifier: NCT02533713 and institutional review board [IRB] ID 919227-45) included participants with and without chronic SCI. Inclusion criteria for participants with SCI were as follows: 18 years of age or older, injury duration of 3 years or more, use of a wheelchair as the primary mode of mobility (more than 50% of the time), SCI level C7–T12, height 155–191 cm, weight less than 113 kg, spasticity in both lower extremities less than 3 on the Modified Ashworth Scale (MAS), and sufficient upper body strength to complete sit-to-sit transfers. Exclusion criteria included enrollment in another clinical trial, pregnancy, orthostatic hypotension with symptomatic fall in blood pressure >30 mm Hg when upright, an active grade 2 or greater pressure ulcer, an unhealed limb or pelvic bone fracture, history of other neurological disease (e.g., stroke, peripheral neuropathy, myopathy), active treatment for epilepsy or thyroid disorders, active use of medications potentially affecting bone health (bisphosphonates, androgenic steroids, estrogenic steroids, lithium, glucocorticoid use for more than 3 months). Participants without SCI were 18 years of age or older and were age, gender, and ethnicity matched to the enrolled participants with SCI. Pregnant women without SCI were excluded.

The second clinical trial (ClinicalTrials.gov Identifier: NCT02946424 and IRB ID 962729-29) included participants with acute SCI (within 3 months of injury) who were 18–60 years of age and who used a wheelchair as their primary mode of mobility (more than 50% of the time). Participants were excluded if they were enrolled in another clinical trial or had contraindications to simvastatin including: drug allergy, history of liver disease, active liver disease (elevated transaminases), moderate or heavy alcohol intake, renal dysfunction (glomerular filtration rate [GFR] of <60 mL/min calculated using the Cockcroft-Gault equation), concurrent use of drugs that cause myopathy or increase the risk of myopathy with simvastatin therapy (gemfibrozil, niacin, cyclosporine, danazol, amiodarone, dronedarone, ranolazine, calcium channel blockers, colchicine), strong CYP34A inhibitors (itraconazole, ketoconazole, posaconazole, voriconazole, erythromycin, clarithromycin, telithromycin, HIV protease inhibitors, boceprevir, telaprevir, nefazodone, cobicistat-containing products), uncontrolled or poorly controlled diabetes (hemoglobin [Hb] A1c >8.0%), or unstable anti-coagulation treatment as indicated by International Normalized Ratio (INR).

Additional exclusion criteria included metabolic bone disease, untreated thyroid disorder, history of bilateral oophorectomy, active use of medications potentially affecting bone health including bisphosphonates, androgenic steroids, estrogenic steroids, anti-epileptics, lithium, glucocorticoid use for more than 3 months, and those who received inhaled glucocorticoids in the past year, and pregnant or lactating women and women of childbearing potential who were unwilling or unable to use a reliable form of contraception. The observational study (IRB ID 1235452-13) included adults with SCI planning to undergo dorsal root entry zone lesioning surgery to alleviate severe neuropathic pain.

For this genome-wide miRNA expression profiling screen study, we studied all participants with banked baseline serum samples. This included 14 participants with acute SCI, 26 participants with chronic SCI, 5 participants with chronic SCI and severe neuropathic pain, and 23 participants without SCI who were sex-and age-matched to the chronic SCI group. We therefore studied a total of 68 participants who completed baseline testing between May 15, 2017 and November 05, 2019. The HealthOne IRB approved all protocols prior to initiation of the study, and all participants gave their written informed consent to participate.

### Variable definition

Information regarding SCI, medical history, health habits, and medication use was obtained by a questionnaire at the time of enrollment. Injury level was considered dichotomously (paraplegia vs. tetraplegia). Age, age at injury, and injury duration were considered as continuous variables. Participants were asked to report a history of medication use including anti-depressants, nonsteroidal anti-inflammatory agents, opioids, gabapentin, and spasmolytics. For all medications, users were defined as those actively taking the drug at the time of testing. Smokers were defined as smoking 20 or more packs of cigarettes or using 336 g (12 oz) of tobacco or more in a lifetime or smoking 1 or more cigarettes a day for at least 1 year. Current smokers reported cigarette use within 1 month of testing. Smoking status was considered dichotomously (current smoker vs. never/past smoker). For active/ever smokers, cigarette exposure was also considered continuously (pack-years). Lifetime alcohol consumption was calculated based on report of average daily, weekly, or monthly quantity and frequency of alcohol consumption. Each glass of wine (4 oz = 10.8 g), beer (12 oz = 13.2 g), and shot of liquor (1.5 oz = 15.1 g) was converted to grams of alcohol.^13,14^ Neuropathic pain was assessed using the International Spinal Cord Injury Pain Data Set.^15,16^ Average pain intensity in the last week (0–10 numerical rating scale), pain interference with mood, pain interference with daily activities, and pain interference with sleep were considered as continuous variables. Neuropathic pain was also considered dichotomously (yes vs. no).

## Motor score

Motor level and completeness of injury were confirmed by physical exam at study entry by the study physician according to the American Spinal Injury Association Impairment Scale (AIS) as previously described.^17,18^ Participants were classified as AIS A (sensory and motor complete, no sensory or motor function below the neurological level of injury), AIS B (motor complete, preservation of sensory but no motor function below the neurological level of injury), or AIS C (motor incomplete, sensory and motor function preserved below the neurological level, and more than half of key muscles below the neurological level are not strong enough to overcome gravity).

### Biochemical analyses

Plasma samples were drawn into an EDTA tube and immediately delivered to the core blood research laboratory at our facility. The samples were centrifuged for 15 min at 2600 rpm (1459 × *g*) at 40℃ and stored at −80℃ until batch analysis. All miRNA analyses were performed at LC Sciences (Houston, TX, USA).

### miRNA bioinformatics pipeline

**FIG. 1.**
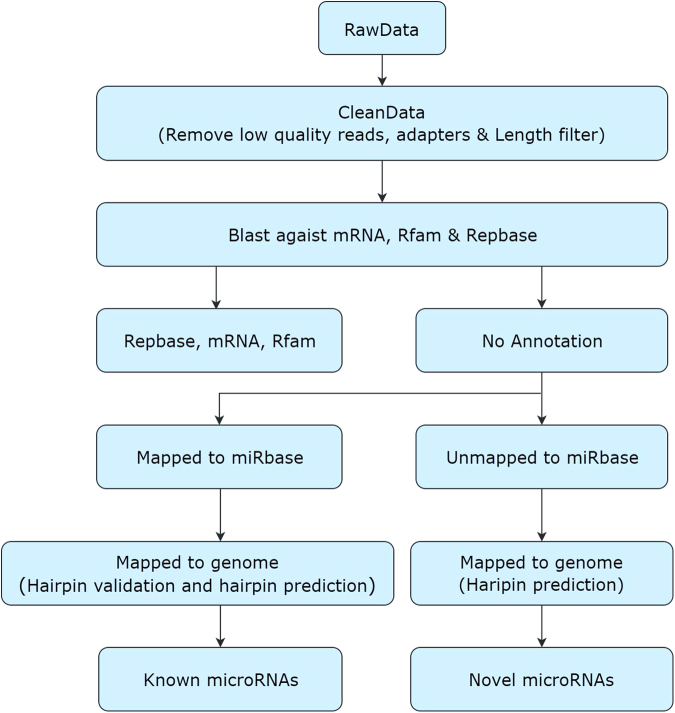
MicroRNA bioinformatics pipeline.

Briefly, comprehensive miRNA/small RNA sequencing service included sample Quality Control (QC), library preparation, and sequencing (50 base pair sequencing, on average a minimum of 7–10 million reads per sample) (Fig. 1). Raw reads were subjected to an in-house program, ACGT101-miR (LC Sciences, Houston, TX, USA) to remove adapter dimers, junk, low complexity, common RNA families (rRNA, tRNA, snRNA, snoRNA), and repeats. Subsequently, unique sequences with length of 18 to 26 nucleotides were mapped to specific species precursors in miRBase 21.0 by BLAST search to identify known miRNAs and novel 3p- and 5p-derived miRNAs. Length variation at both 3’ and 5’ ends and one mismatch inside of the sequence were allowed in the alignment. The unique sequences mapping to specific species of mature miRNAs in hairpin arms were identified as known miRNAs. The unique sequences mapping to the other arm of known specific species precursor hairpin opposite to the annotated mature miRNA-containing arm were considered to be novel 5p- or 3p-derived miRNA candidates. The remaining sequences were mapped to other selected species precursors (with the exclusion of specific species) in miRBase 21.0 by BLAST search, and the mapped pre-miRNAs were further BLASTed against the specific species genomes to determine their genomic locations. The unmapped sequences were BLASTed against the specific genomes, and the hairpin RNA structures containing sequences were predicated from the flank 80 nt sequences using RNAfold software. 

The criteria for secondary structure prediction were: 1) number of nucleotides in one bulge in stem (≤12); 2) number of base pairs in the stem region of the predicted hairpin (≥16); 3) cutoff of free energy (kCal/mol less than or equal to -15); 4) length of hairpin (up and down stems + terminal loop ≥50); 5) length of hairpin loop (≤20); 6) number of nucleotides in one bulge in mature region (≤8); 7) number of biased errors in one bulge in mature region (≤4); 8) number of biased bulges in mature region (≤2); 9) number of errors in mature region (≤7); 10) number of base pairs in the mature region of the predicted hairpin (≥12); and 11) percent of mature in stem (≥80).

Raw data were filtered using a filter module in an in-house program, ACGT101-miR, to delete low-quality reads, 3' adapter sequences, and contaminations. The sequences ≥18 nt of clean data were annotated in the Rfam database to remove non-coding RNA (rRNA, tRNA, snRNA, snoRNA) and degradation fragments of mRNA. The remaining sequences were aligned against an miRNA database, miRbase (Release 22), and perfectly matched sequences were considered conserved *Homo sapiens* (hsa) miRNAs. After processed by filter module in ACGT101-miR, unique reads were analyzed using ACGT101-miR to detect conserved and novel hsa miRNAs. Normalization of sequence counts in each sample (or data set) was achieved by dividing the counts by a library size parameter of the corresponding sample. The library size parameter is a median value of the ratio between the counts of a specific sample and a pseudo-reference sample. A count number in the pseudo-reference sample is the count geometric mean across all samples.

$$S_{j}={\mathrm{median}}_{i}\left(\frac{c_{ij}}{{\left(\prod_{k=1}^{m}c_{ik}\right)}^{1∕m}}\right)$$

where Sj is the library size parameter; cij is the count number of sequence i of sample j; m is the total number of samples involved.

### Target prediction analysis

Target genes potentially regulated by miRNAs of interest were predicted using the consensus of three publicly available miRNA target databases: TargetScan, MicroCosm, and miRTarBase. The predicted target false-positive rate was reduced significantly by applying a cutoff of −0.4 in the context scores for TargetScan results; each miRNA target was cross-referenced against this gene set. Enrichment and pathway analysis of predicted miRNA gene targets was undertaken using MetaCoreTM process networks, pathway maps, and GO molecular functions/processes. Top pathways were ranked based on z-score and the additional enriched gene count per pathway. Interactions between miRNAs and their target gene networks for each tissue were visualized using CyTargetLinker v3.0.1, an open source software package for Cytoscape v.4.0.43.

Circos v0.67 was used to display interaction between miRNAs and target genes in a circular layout, facilitating the visualization of the position of the miRNAs and target genes in the respective genome; only target genes passing the described cutoff were visualized in the CircosPlot. hsa-miR-19a-3p and hsa-miR-19b-3p from an experimental data set were loaded. yFiles Organic Layout was performed to better visualize physical and genetic interaction networks. Targets with strong experimental evidence for hsa-miR-19a-3p and hsa-miR-19b-3p were retrieved, following which, an extended literature search of the PubMed database was performed for further confirmation of neuro-associated validated targets.

### Statistical analysis

We conducted two screenings: one based on presence or absence of SCI (No SCI *n* = 23, acute SCI *n* = 14, and chronic SCI *n* = 26) and one based on severe neuropathic pain in chronic SCI (*n* = 5, pain score range 8–10) versus no neuropathic pain in chronic SCI (*n* = 4). For each comparison, normalized deep-sequencing counts were analyzed by selectively using Fisher's exact test, χ^2^ 2X2 test, χ^2^ nXn test, Student's *t* test, or analysis of variance (ANOVA) as appropriate. Because False Discovery Rate (FDR) adjusted *p*-values may be too conservative for screening studies, we determined differential expression based on log twofold concentration change and raw *p*-value <0.05. This study is adequately powered to detect twofold differences in expression between groups.^19^ All subsequent analyses were performed using SAS 9.4 (SAS Institute, Inc., Cary, NC, USA). To compare subject characteristics as appropriate, *t* tests or χ^2^ tests were used. General linear models (PROC GLM) were then applied including all participants with clinical/demographic data and miRNA values (*n* = 66, Table 1) to confirm the screening findings. We assessed associations between biomarker levels (hsa-miR-19a-3p and hsa-miR-19b-3p), pain, and pain interference scores. Pain interference with mood was selected for multi-variable model building based on univariate findings. Factors with a *p-*value of <0.10 in the univariate models were included in the multi-variable models of pain interference with mood. Factors with a *p-*value of <0.05 were considered statistically significant.

**Table 1.** Characteristics of Confirmatory Cohort Based on Presence of Neuropathic Pain

Variable	No neuropathic pain (*n* = 38)	Neuropathic pain (*n* = 28)	Total (*n* = 66)	*P*
Demographics				
Age (years) [mean ± SD]	36.62 ± 10.78	40.11 ± 12.77	38.10 ± 11.70	0.86
Male, *n* (%)	24 (63.1)	24 (85.7)	48 (72.7)	0.04

Injury characteristics				
AIS classification				
• A, *n* (%)	10 (66.7)^a^	20 (71.4)	30 (69.8)	0.60
• B, *n* (%)	4 (26.7)^a^	4 (14.3)	8 (18.6)	
• C, *n* (%)	1 (6.6)^a^	4 (14.3)	5 (11.6)	
Tetraplegia, *n* (%)	4 (26.7)^a^	6 (21.4)	10 (23.3)	0.71
Injury duration (years) [mean ± SD]	6.67 ± 8.10^a^	8.81 ± 9.06	8.06 ± 8.70	<0.0001
Age at injury (years) [mean ± SD]	28.73 ± 11.41^a^	30.39 ± 12.44^e^	29.73 ± 11.91	0.003

Medication use				
Active opioid use, *n* (%)	4 (10.5)	8 (34.8)^e^	12 (19.7)	0.04
Active spasmolytic use, *n* (%)	6 (15.8)	10 (43.5)^e^	16 (26.2)	0.01
Active gabapentin use, *n* (%)	3 (7.9)	10 (43.5)^e^	13 (21.3)	0.002
Active antidepressant use, *n* (%)	1 (2.6)	2 (8.7)^e^	3 (4.9)	0.55

Health habits				
Current alcohol user, *n* (%)	32 (86.5)^b^	19 (90.5)^f^	51 (87.9)	1.0
Lifetime alcohol exposure kg/years [mean ± SD]	147.78 ± 205.11^c^	116.27 ± 97.21^c^	136.04 ± 172.40	0.23
Current smoker, *n* (%)	1 (2.7)^b^	4 (19.1)^f^	5 (8.6)	0.05
Cigarette exposure (pack-years) [mean ± SD]	9.67 ± 9.88^d^	6.21 ± 5.03^d^	7.56 ± 7.24	0.005

^a^ Among SCI *n* = 15; ^b^available data for *n* = 37; ^c^among ever alcohol users; ^d^among ever smokers; ^e^available data for *n* = 23; ^f^available data for *n* = 21.

AIS, American Spinal Injury Association Impairment Scale; SCI, spinal cord injury; SD, standard deviation.

## Results

### Subject characteristics of genome-wide microRNA expression profile screenings

Subject characteristics are presented in Table 2A,B. For the SCI versus No SCI screening (Table 2A), participants with No SCI were 37.1 ± 11.1 years of age (range 24.2 to 57.9 years) and 57% male. None reported neuropathic pain. Participants with acute SCI were 37.0 ± 10.8 years of age (range 21.2 to 54.7 years), 79% male, and 2.9 ± 0.7 months post-injury (range 1.7 to 4.4 months). Fifty-four percent reported neuropathic pain. Participants with chronic SCI were 36.5 ± 11.0 years of age (range 18.6 to 70.6 years), 77% male, and 10.4 ± 6.6 years post-injury (range 3.1 to 23.4 years). Sixty-five percent reported neuropathic pain. All participants with SCI used a wheelchair as their primary mode of mobility. Those with acute SCI were more likely to be tetraplegic than those with chronic SCI. Medication use (opioids, spasmolytics, and gabapentin) was more common in acute SCI than chronic SCI or No SCI. For the neuropathic pain in SCI screening (Table 2B), those with neuropathic pain in SCI were all males, 50.2 ± 16.9 year of age (range 30.2 to 72.0 years) and 16.6 ± 14.0 years post-injury (range 3.5 to 33.3 years). SCI without neuropathic pain were 75% male, 30.1 ± 6.7 years of age (25.2 to 40.0 years), and 8.0 ± 6.1 years post-injury (3.6 to 17.1 years).

**Table 2A.** Characteristics of Genome-Wide MicroRNA Expression Profile Screening

Variable	No SCI (*n* = 23)	Acute SCI (*n* = 14)	Chronic SCI* (n* = 26)	Total (*n* = 63)	*P*
Demographics					
Age (years) [mean ± SD]	37.08 ± 11.07	36.95 ± 10.77	36.54 ± 10.97	36.83 ± 10.79	0.98
Male, *n* (%)	13 (56.5)	11 (78.6)	20 (76.9)	44 (69.8)	0.21
Pain, *n*(%)	11 (47.8)	13 (100.0)^a^	21 (80.8)	45 (72.6)	0.001
Neuropathic pain, *n* (%)	0 (0.0)	7 (53.8)^a^	17 (65.4)	24 (38.7)	<0.0001

Injury characteristics					
AIS classification	N/A				
• A, *n* (%)		10 (71.4)	17 (65.4)	27 (67.5)	0.88
• B, *n* (%)		3 (21.4)	5 (19.2)	8 (20.0)	
• C, *n* (%)		1 (7.2)	4 (15.4)	5 (12.5)	
Tetraplegia, *n* (%)	N/A	8 (57.1)	2 (7.7)	10 (25.0)	0.001
Injury duration (years) [mean ± SD]	N/A	0.24 ± 0.06	10.35 ± 6.64	6.81 ± 7.21	<0.0001
Age at injury (years) [mean ± SD]	N/A	36.21 ± 10.70	25.76 ± 10.69	29.42 ± 11.70	0.005

Biomarkers					
MiR-19a (normalized deep sequencing count) [mean ± SD]	1243.83 ± 820.64	1671.36 ± 815.03	989.53 ± 642.25	1233.89 ± 782.86	0.02
MiR-19b (normalized deep sequencing count) [mean ± SD]	2573.04 ± 1440.92	3961.14 ± 1833.18	2100.00 ± 1137.60	2686.29 ± 1574.51	0.0009

Medication use					
Active opioid use, n(%)	0 (0.0)	5 (35.7)	7 (26.9)	12 (19.1)	0.003
Active spasmolytic use, *n* (%)	0 (0.0)	10 (71.4)	6 (23.1)	16 (25.4)	<0.0001
Active gabapentin use, *n* (%)	1 (4.4)	8 (57.1)	4 (15.4)	13 (20.6)	0.0008
Active antidepressant use, *n* (%)	0 (0.0)	1 (7.1)	2 (7.7)	3 (4.7)	0.43

Health habits					
Current alcohol user, *n* (%)	23 (100.0)	9 (90.0)^b^	20 (76.9)	52 (88.1)	0.03
Lifetime alcohol exposure kg/years [mean ± SD]	120.54 ± 109.56	72.71 ± 81.86^c^	180.42 ± 239.39^c^	135.30 ± 170.78	0.25
Current smoker, *n* (%)	0 (0.0)	2 (20.0)^b^	3 (11.5)	5 (8.5)	0.10
Cigarette exposure (pack-years) [mean ± SD]	N/A	12.51 ± 7.65^d^	3.27 ± 3.72^d^	7.16 ± 7.24	0.01

^a^ Available data for *n* = 13; ^b^available data for *n* = 10; ^c^among ever alcohol users; ^d^among ever smokers.

SD, standard deviation.

**Table 2B.** Characteristics of Genome-Wide MicroRNA Expression Profile Screening Neuropathic Pain Cohort

Variable	No neuropathic pain (*n* = 4)	Neuropathic pain (*n* = 5)	Total (*n* = 9)	*P*
Demographics				
Age (years) [mean ± SD]	30.13 ± 6.72	50.16 ± 16.94	41.26 ± 16.49	0.06
Male, *n* (%)	3 (75.0)	5 (100.0)	8 (88.9)	0.44

Injury characteristics				
AIS classification				
• A, *n* (%)	3 (75.0)	5 (100.0)	8 (88.9)	0.44
• B, *n* (%)	1 (25.0)	0 (0.0)	1 (11.1)	
Tetraplegia, *n* (%)	0 (0.0)	0 (0.0)	0 (0.0)	N/A
Injury duration (years) [mean ± SD]	8.03 ± 6.13	16.62 ± 14.01	12.80 ± 11.52	0.29

Biomarkers				
MiR-19a (normalized deep sequencing count) [mean ± SD]	857.50 ± 390.35	3174.80 ± 1079.10	2144.89 ± 1459.80	0.006
MiR-19b (normalized deep sequencing count) [mean ± SD]	1791.75 ± 772.43	7005.20 ± 1873.36	4688.11 ± 3086.83	0.001

AIS, American Spinal Injury Association Impairment Scale; SD, standard deviation.

### Differential expression of hsa-miR-19a-3p and hsa-miR19-b-3p levels

**FIG. 2.**
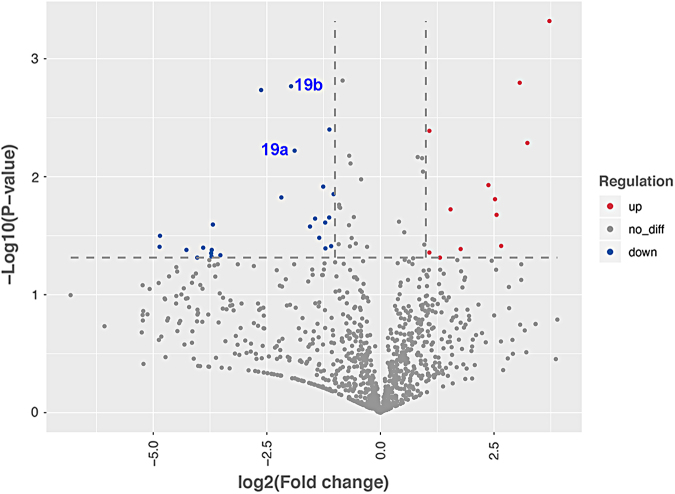
Volcano plot comparing Log2 (fold change) of miRNA expression values for participants who have a chronic SCI reporting no neuropathic pain (*n* = 4) versus those who have chronic SCI and report neuropathic pain (*n* = 5). Significantly upregulated genes shown in red, significantly downregulated genes shown in blue (*p* < 0.05). Some dots may represent more than one data point. Refer to Table 2B for participants characteristics. 19a: hsa-miR-19a-3p; 19b: hsa-miR-19b-3p. miRNA, microRNA; SCI, spinal cord injury.

**FIG. 3.**
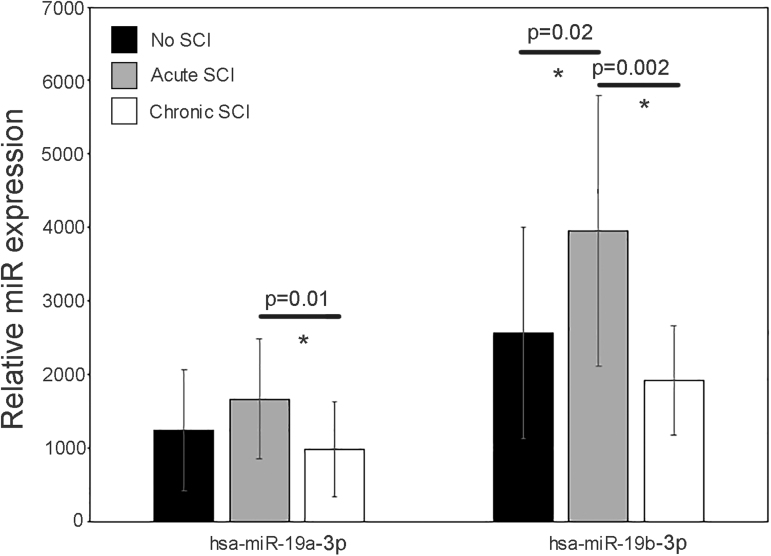
Expression values of hsa-miR-19a-3p and hsa-miR-19b-3p in participants with no SCI (black bars, mean expression value), participants who have an acute SCI (gray bars, mean expression value), and participants who have a chronic SCI (white bars, mean expression value). Black horizontal lines indicate the *p*-values for the differences between groups. Error bars are SD. Asterisks (*) indicate significant differences between groups. SCI, spinal cord injury; SD, standard deviation.

**FIG. 4.**
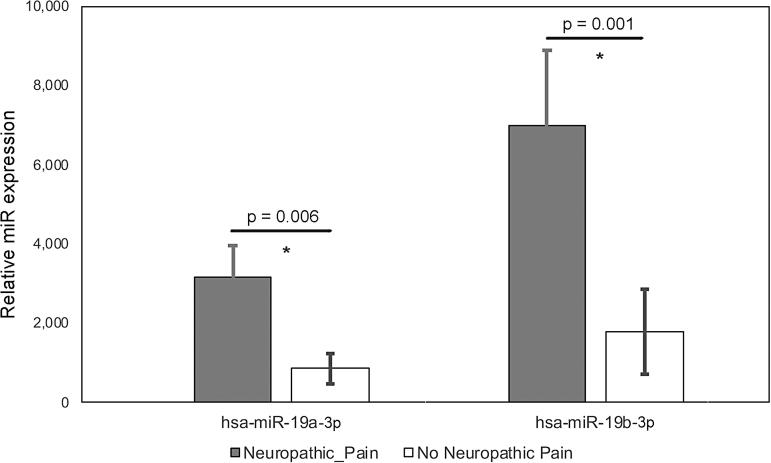
Expression values of hsa-miR-19a-3p and hsa-miR-19b-3p for participants who have a chronic SCI reporting no neuropathic pain (white bars. mean expression value) versus those who have chronic SCI and report neuropathic pain (gray bars: mean expression value). Black horizontal lines indicate the *p*-values for the differences between groups. Error bars are SD. Asterisks (*) indicate significant differences between groups. SCI, spinal cord injury; SD, standard deviation.

We detected 2367 distinct miRNAs. In acute SCI (146) or chronic SCI (237) versus No SCI, 383 miRNAs were significantly (*p* < 0.05) up- or downregulated. In acute SCI versus chronic SCI, 208 miRNAs were significantly (*p* < 0.05) up- or downregulated. In participants with chronic SCI reporting no neuropathic pain versus those reporting neuropathic pain, 71 miRNAs (Fig. 2, Table 2B) were significantly (*p* < 0.05) up or downregulated. We selected hsa-miR-19a-3p and hsa-miR-19b-3p for further analysis based on log twofold concentration change, *p*-value, and their reported role in neuropathic pain and neuroinflammation.^20^ One or both were differentially expressed in acute SCI versus No SCI (Fig. 3, 3961 vs. 2573, *p* = 0.02 for 19b), acute SCI versus chronic SCI (Fig. 3, 1671> vs. 990, *p* = 0.01 for 19a and 3961 vs. 2100, *p* = 0.002 for 19b), and neuropathic pain versus no neuropathic pain (Fig. 4, 3175 vs. 857, *p* = 0.006 for 19a and 7005 vs. 1792, *p* = 0.001 for 19b).

### Validated targets of miR-19a and miR-19b

**FIG. 5.**
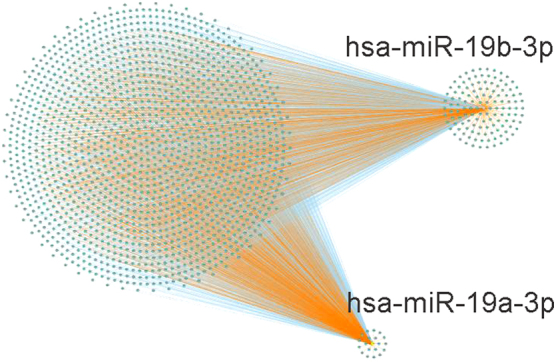
microRNA-target interaction network generated by Cytoscape software demonstrating the density of shared and unique targets for each microRNA. Targets with strong experimental evidence for hsa-miR-19a-3p and hsa-miR-19b-3p were retrieved from miRTarBase. Blue color indicates targets shared by hsa-miR-19a-3p and hsa-miR-19b-3p, and yellow color indicates targets specific for either hsa-miR-19a-3p or hsa-miR-19b-3p.

Targets with strong experimental evidence for hsa-miR-19a-3p and hsa-miR-19b-3p were retrieved from miRTarBase (Fig. 5), following which, we performed an extended literature search of the PubMed database and further confirmed those neuro-associated validated targets. miR-19a and miR-19b representative validated target genes of miR-19a and miR-19b are shown in Table 3A–C. Phosphatase and Tensin Homolog (PTEN),^21,22^ Rap Guanine Nucleotide Exchange Factor (2Rapgef2),^23^ voltage-gated potassium channel subunits,^20^ Tumor Protein p53 (p53),^24^ and RUNX Family Transcription Factor 3 (RUNX3)^25^ are common targets shared by both miR-19a and miR-19b. Further, miR-19a has its own validated targets including Suppressor of Cytokine Signaling 1 (SOCS1), Methyl-CpG Binding Protein 2 (MeCP2), Ras Homolog Family Member B (RhoB), Peroxisome Proliferator Activated Receptor Alpha (PPARα), and Leucine Rich Repeats and Immunoglobulin Like Domains 1 (LRIG1).^26^ miR-19b also has its confirmed targets, FMR1 Autosomal Homolog 1 (FXR1)^27^ and Signal Transduction and Activator of Transcription 3 (STAT3).^28^ Among these validated targets, especially, SOCS1 was confirmed as a direct target of miR-19a in neuropathic pain rat models.^29^ Also, miR-19a and miR-19b regulate their common shared target, multiple functionally related voltage-gated potassium channel in chronic neuropathic pain in rats.^20^

**Table 3A.** Validated Targets of Both MiR-19a and 19b

Target	Model	Tissue	MicroRNA effect	Relative expression	References
Phosphatase and Tensin Homolog (PTEN)^*^	*In vitro* Human *In vitro* Rat	Malignant glioma cell lines Resected glioma SH-SY5Y cells SCI	Proliferation Oncogenic Anti-apoptotic Anti-apoptotic	↑ Gliomagenesis ↓ SCI	Jia et al. Xu et al.
Rap Guanine Nucleotide Exchange Factor (2Rapgef2^*^)	*In vitro*	Neural progenitor cell (NPC)	Regulates cell migration	↑ NPCs ↓ During neuronal development	Han et al.
Voltage-gated potassium channel subunits^*^	Rat	Primary sensory neurons	Regulate A-type outward potassium currents	↑ Mechanical allodynia	Sakai et al.
Tumor Protein p53 (p53)	*In vitro* Rat	SH-SY5Y cells Al-malt induced apoptosis in rat brain	Anti-apoptotic Anti-apoptotic	↑ Al-induced neural cell apoptosis ↑ Al-induced neural cell apoptosis	Zhu et al.
RUNX Family Transcription Factor 3 (RUNX3)	*In vitro* *Mouse* Human	U87 and LN229 glioma cell lines Resected glioma Resected glioma	Inhibition of proliferation and invasion Apoptotic Anti-oncogenic	↓ Glioma cell lines ↑ Mice implanted with As-miR-19a/b in right forebrain ↓ Gliomagenesis	Sun et al.

SCI, spinal cord injury.

**Table 3B.** Validated Targets of MiR-19a

Target	Model	Tissue	MicroRNA effect	Relative expression	References
Suppressor of Cytokine Signaling 1 (SOCS1)	Rat bilateral chronic constriction injury	Sciatic nerve	Anti-inflammatory	↑ Neuropathic pain	Wang et al.
Methyl-CpG Binding Protein 2 (MeCP2)	*In vitro* Mouse	Neuro-2a cells Dorsal root ganglia	N/A		Manners et al.
Ras Homolog Family Member B (RhoB)	*In vitro* Human	LN18, LN229, LN428, SW1783, SW1088, U251, U373, and U87 glioma cell lines Resected glioma	Promotion of proliferation and invasion Oncogenic	↑ Following miRNA-19a knockdown ↓ With miRNA-19a N/A (They didn't check RhoB expression at mRNA or protein level in resected glioma. They just checked miR-19a expression.)	
Peroxisome Proliferator Activated Receptor Alpha (PPARα)	Human *In vitro* Mouse	Resected glioma U87 and LN229 glioma cells Resected glioma	Anti-oncogenic Inhibition of glioma cell colony formation, invasion, and glucose consumption Anti-oncogenic	↓ Gliomagenesis ↑ Following miRNA-19a knockdown ↓ Gliomagenesis	Shi et al.
Leucine Rich Repeats and Immunoglobulin Like Domains 1 (LRIG1)	Human *In vitro*	Resected glioma U251, U87, A172, and U118 glioma cell lines	Oncogenic Inhibition of glioma cell proliferation	N/A (They didn't check LRIG1 expression at mRNA or protein level in resected glioma. They just checked miR-19a expression.) ↑ Following miRNA-19a knockdown	Shao et al.

**Table 3C.** Validated Targets of MiR-19b

Target	Model	Tissue	MicroRNA effect	Relative expression	References
FMR1 Autosomal Homolog 1 (FXR1 )	*In vitro*	SH-SY5Y cells	Apoptotic	↑ Fragile X syndrome	Ma et al.
Signal Transduction and Activator of Transcription 3 (STAT3)	*In vitro*	SH-SY5Y cells	Increase in STAT3 phosphorylation	**↓** Alzheimer's disease	Wu et al.

### Discriminative ability of hsa-miR-19a-3p and hsa-miR-19b-3p to distinguish between pain and no pain

**FIG. 6.**
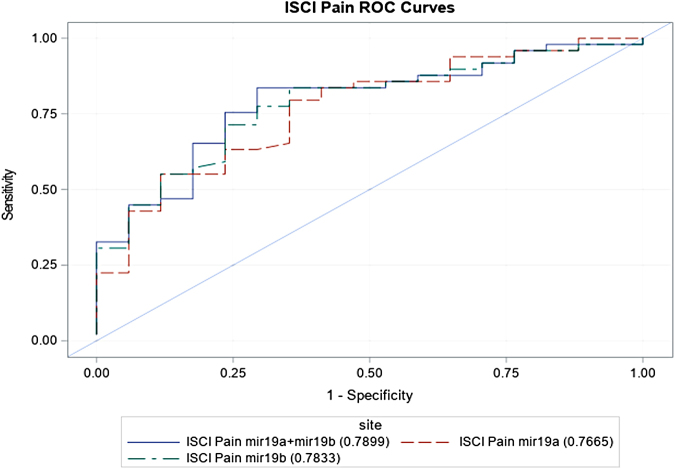
ROC curve of: hsa-miR-19a-3p (red dotted line), hsa-miR-19b-3p (green dotted line), and hsa-miR-19a-3p and hsa-miR-19b-3p (blue solid line) as a predictor of ISCI pain in participants who have SCI. AUC for hsa-miR-19a-3p is 0.7665 (*p* = 0.0174), for hsa-miR-19b-3p is 0.7833 (*p* = 0.0109), and for hsa-miR-19a-3p and hsa-miR-19b-3p together is 0.7899 (*p* = 0.0389). AUC, area under the curve; ISCI, International Spinal Cord Injury; ROC, receiver operating characteristic; SCI, spinal cord injury.

Subject characteristics based on the presence (*n* = 28) or absence (*n* = 38) of neuropathic pain are presented in Table 1. The neuropathic pain group was more likely to be male (86% vs. 63%, *p* = 0.04), to have been injured at an older age (30 vs. 29 years, *p* = 0.003), to have longer injury duration (9 vs. 7 years, *p* < 0.0001), and to use medications (opioids, *p* = 0.04, spasmolytics, *p* = 0.01 and gabapentin, *p* = 0.002) than the no pain group. Both hsa-miR-19a-3p (area under the curve [AUC] 0.77) and hsa-miR-19b-3p (AUC 0.78) have moderate to strong discriminative ability to distinguish between those with and without pain. The discriminative ability increased when considering both together in the same model (AUC 0.79, Fig. 6).

### Univariate factors associated with neuropathic pain interference with mood

In univariate analyses (Table 4), men reported greater pain interference with mood than women, active opioid users reported greater interference than non-users, and active spasmolytic users reported greater interference than non-users. hsa-miR-19a-3p and hsa-miR-19b-3p levels were both positively associated with pain interference with mood. We also found a positive association between hsa-miR-19a-3p and hsa-miR-19b-3p levels and pain interference with activities of daily living and pain interference with sleep (data not shown, *p* = 0.0003 to 0.03).

**Table 4.** Univariate Predictors of Neuropathic Pain Interference with Mood

Continuous variable	β ± SE	*P*
Age (unit pain interference score/year)	0.03 ± 0.02	0.29
Injury duration (unit pain interference score/year)	0.07 ± 0.04^a^	0.14
Age at injury (unit pain interference score /year)	-0.007 ± 0.03^a^	0.84
MiR-19a (unit pain interference score/normalized deep sequencing count)	0.001 ± 0.0003	0.003
MiR-19b (unit pain interference score/normalized deep sequencing count)	0.0005 ± 0.0001	0.0004
Lifetime alcohol use (unit pain interference score/kg/year)	0.002 ± 0.002^b^	0.31
Cigarette exposure (unit pain interference score/pack-year)	-0.05 ± 0.09^c^	0.61

Categorical variable	Mean pain interference score ± SE	P
Sex		0.03
• Male	2.83 ± 2.82	
• Female	1.16 ± 2.25	
Injury level		0.22
• Paraplegia	3.57 ± 3.01^a^	
• Tetraplegia	2.30 ± 2.11	
Active opioid use		0.005
• Yes	3.83 ± 2.85^d^	
• No	1.59 ± 2.28	
Active spasmolytic use		0.03
• Yes	3.25 ± 2.88^d^	
• No	1.60 ± 2.29	
Active gabapentin use		0.16
• Yes	2.92 ± 2.62^d^	
• No	1.79 ± 2.49	
Alcohol use		0.96
• Yes	1.96 ± 2.46^e^	
• No	2.00 ± 3.21	
Smoker status		0.61
• Ever	2.17 ± 2.69^e^	
• Never	1.82 ± 2.45	

^a^ Among SCI *n* = 43; ^b^among ever alcohol users; ^c^among ever smokers^; d^available data for *n* = 61; ^e^available data for *n* = 5.8.

SCI, spinal cord injury; SE, standard error of the mean.

### Multi-variable factors associated with neuropathic pain interference with mood

In multi-variable models (data not shown) hsa-miR-19a-3p and hsa-miR-19b-3p were no longer associated with pain interference with daily activities or sleep. In separate multi-variable models (Table 5), sex, spasmolytic use, and miR-19a were no longer significantly associated with pain interference with mood. Opioid users had significantly greater pain interference than non-users (3.7 vs. 1.6, *p* = 0.008). Pain interference with mood score increased by 0.0004 ± 0.0001 units for every 1 unit increase in hsa-miR-19b-3p (*p* = 0.04). This model explained 18% of the variation in pain interference with mood score (model *p* = 0.003).

**Table 5.** Multi-Variable Factors Associated with Neuropathic Pain Interference with Mood

Model* p* = 0.003,* R^2^* = 0.18		
Continuous variable	β ± SE	*P*
MiR-19b (normalized deep sequencing count)	0.0004 ± 0.0001	0.04

Categorical variable	Mean pain interference score ± SE	P
Active opioid use		0.008
• Yes	3.71 ± 0.67	
• No	1.62 ± 0.33	

SE, standard error of the mean.

## Discussion

In this study we performed an untargeted genome-wide miRNA screening to study differential miRNA expression in plasma from 68 healthy, community-dwelling adults with and without SCI. We found 383 miRNAs that were differentially expressed in acute SCI or chronic SCI versus no SCI. In addition, we found 71 miRNAs were differentially expressed in chronic neuropathic pain versus no neuropathic pain. In a subsequent analysis, we selected hsa-miR-19a-3p and hsa-miR-19b-3p based on *p*-value, fold change, and their known role as regulators of neuroinflammation and neuropathic pain. We found that both hsa-miR-19a-3p and hsa-miR-19b-3p levels are significantly higher in acute SCI compared with chronic SCI and in those with chronic SCI with neuropathic pain versus those without neuropathic pain. hsa-miR-19b-3p levels are also significantly greater in both acute and chronic SCI versus No SCI. Additionally, both hsa-miR-19a-3p and hsa-miR-19b-3p have moderate to strong discriminative ability to distinguish between those with and without pain. We also found that after adjusting for opioid use, hsa-miR-19b-3p is positively associated with pain interference with mood. Our large sample size is a strength of this study as it reduces the risk of small sample size error.^19^

Neuropathic pain presents at or below the level of injury^30,31^ and is cited as the most “severe pain” post-SCI.^32^ Unmitigated pain limits physical activity, negatively impacts rehabilitation, limits work and social activities, and reduces quality of life.^33^ Unfortunately, neuropathic pain is considered refractory to most available treatments, likely because treatment is offered after pain has already developed. It is possible that substantial and perhaps irreversible neuronal changes have already occurred, limiting the therapeutic potential. Therefore, early preventative strategies to mitigate chronic neuropathic pain after SCI may be more effective. Development of and validation of biomarkers might help to identify those most at risk of chronic sublesional neuropathic pain and therefore who might benefit from preventative strategies, both pharmacological and non-pharmacological.^34^

A poor understanding of the physiological basis of neuropathic pain continues to hinder the identification of novel, effective interventions. The study of miRNA in neuropathic pain is a relatively new field of research. However, the significance of miRNA alterations in a variety of rodent pain models and in clinical conditions characterized by pain has been clearly established. miRNAs exert post-transcriptional modulation of large sections of the genome by binding to regulatory gene elements and inhibiting the translation of many genes.^35,36^ Significant alterations in miRNAs expression (together with the resultant changes in protein expression) have been reported in both the affected tissues and in blood from patients suffering from several pain conditions such as complex regional pain syndrome, cystitis-induced chronic pain, and irritable bowel disorder. They are found in every human tissue and biofluid, are resistant to RNAse degradation, and have the ability to cross the blood–brain barrier, making them excellent candidate biomarkers for neurotrauma-related conditions, neurorecovery, and response to various neuroprotective interventions.^37^

Diverse causes of neuropathic pain are associated with excessive inflammation in both the PNS and CNS, which may contribute to the initiation and maintenance of persistent pain. Chemical mediators, such as cytokines, chemokines, and lipid mediators, released during an inflammatory response have the undesired effect of sensitizing and stimulating nociceptors, their central synaptic targets, or both. Although there is lack of consensus in the literature regarding male and female specific pain pathways, sex dimorphism has been reported in the development and maintenance of neuropathic pain.^38^ This includes sex differences in immune-mediated pain and the effect of sex hormones on nociception and morphine tolerance.^38^ In our study men reported higher levels of pain interference with mood in univariate analyses. However, sex was no longer significantly associated with pain interference with mood in multi-variable models. Caution must be taken when interpreting this result as our sample included too few women to make meaningful comparisons based on sex. Understanding the potential impact of sex on these processes better may lead to potentially novel therapeutic targets for neuropathic pain.

There are many validated targets of hsa-miR-19a-3p and hsa-miR-19b-3p, including SOCS1.^39^ Members of the SOCS family are thought to modulate neuroinflammation.^40^ In particular, two members, SOCS1 and SOCS3, control inflammatory cytokine signaling in neurons,^41–43^ Schwann cells,^41,44^ oligodendrocytes,^45,46^ astrocytes,^47,48^ and microglia.^49–51^ Expression of SOCS1 and SOCS3 by microglia is associated with reduced nitric oxide (NO) production and decreased sensitivity to cytokine-induced signaling, thereby reducing neuroinflammation.^52,53^ Reduced SOCS1 expression in microglia, on the other hand, promotes pro-inflammatory microglia and aggravation of neuroinflammation.^54^ Upregulation of these miRNAs is associated with increased inflammation. miR-19a-3p and miR-19b-3p may contribute to neuroinflammation via modulation of SOCS1 expression after SCI, thereby providing a potential mechanistic link between neuroinflammation and neuropathic pain. miR-19a-3p and miR-19b-3p both belong to the miR-17-92 cluster located on human chromosome 13. miRNA clusters result from genome duplication and are classified based on sequence homology. Overexpression of miR-17-92 cluster members is associated with neuropathic pain in a variety of models and clinical contexts, and this is thought to be due to regulation of voltage-gated potassium channels (Table 3).^20^

Our findings are consistent with prior reports and support a role for the coordinated regulatory effect of miR-17-92 cluster miRNAs on multiple voltage-gated potassium channels in the development and/or maintenance of neuropathic pain. Several studies have demonstrated alterations in miRNA levels due to therapeutic interventions, including exercise and medications. This suggests that miRNAs are therapeutic targets. In fact, miR-19-3p levels have been shown to change in response to exercise, folic acid, and resveratrol.^55^

SOCS1 was found to play a role in inflammatory reactions by regulating JAK/STAT signaling pathways in innate immune cells and non-immune cells^56–59^ JAK/STAT blockade in spinal cord microglial cells and astrocytes can attenuate local inflammation and pain hypersensitivity.^60,61^ Interestingly, SOCS3 can also block JAK/STAT3 activity, preventing the abnormal expression of interleukin (IL)-6, CC chemokine ligand CCL2, and activating transcription factor ATF3 induced in the spinal cord by chronic constriction injury (CCI) of the sciatic nerve and substantially attenuated mechanical hypersensitivity (allodynia) in rats. Qin and colleagues^51^ reported interferon (IFN)-induced SOCS1 negatively regulates CD40 gene expression in macrophages and microglia. They found IFN-induced expression of the SOCS1 protein, which functioned in a negative regulatory feedback loop to inhibit IFN signaling, and ultimately CD40 expression.^51^ In addition to SOCS1 and SOCS3, voltage-gated potassium channel subunits as the validated target of both miR-19a-3p and miR-19b-3p provided the opportunity to develop a novel analgesic strategy for therapy for neuropathic pain based on concurrent regulation of multiple functionally related proteins. Overexpression of miR-19a, miR-19b, miR-18, and miR-92a cluster members elicits mechanical allodynia in rats, whereas their blockade alleviates mechanical allodynia in a rat model of neuropathic pain.^20^ Taken together, these results strongly support a role for miR-19a-3p and miR-19b-3p in the pathogenesis of neuropathic pain.

There are several limitations of the current study to consider. Despite being a comparatively large sample for miRNA expression profile screening studies, the candidate miRNAs identified in this study should be confirmed in a larger, more heterogeneous sample. Moreover, this is a cross-sectional study and therefore we cannot assign causality to any of the identified relationships. Mechanistic studies focused on these miRNAs, SOCS1/SOCS3 pathways, and inflammatory cytokines are needed. Despite these limitations, this expression profile screening study provides important insight into the potential role of two miRNAs and neuropathic pain in SCI.

## Abbreviations Used

2Rapgef2: Rap Guanine Nucleotide Exchange Factor
AIS: American Spinal Injury Association Impairment Scale
ANOVA: analysis of variance
AUC: area under the curve
CCI: chronic constriction injury
CNS: central nervous system
FDR: False Discovery Rate
FXR1: FMR1 Autosomal Homolog 1
GFR: glomerular filtration rate
Has: *Homo sapiens*
Hb: hemoglobin
IASP: International Association for the Study of Pain
IFN: interferon
IL: interleukin
INR: International Normalized Ratio
IRB: institutional review board
ISCI: International Spinal Cord Injury
LRIG1: Leucine Rich Repeats and Immunoglobulin Like Domains 1
MAS: Modified Ashworth Scale
MeCP2: Methyl-CpG Binding Protein 2
miRNA: microRNA
mRNA: messenger RNA
NO: nitric oxide
P53: Tumor Protein p53
PNS: peripheral nervous system
PPARα: Peroxisome Proliferator Activated Receptor Alpha
PTEN: Phosphatase and tensin homolog
PTEN: Phosphatase and Tensin Homolog
QC: Quality Control
RhoB: Ras Homolog Family Member B
ROC: receiver operating characteristic
RUNX3: RUNX Family Transcription Factor 3
SCI: spinal cord injury
SD: standard deviation
SE: standard error of the mean
SOCS1: Suppressor of Cytokine Signaling 1
STAT3: Signal Transduction and Activator of Transcription 3
UTR: untranslated region
